# Rapid detection of alveolar echinococcosis in hepatic nodules of horses by recombinase polymerase amplification assay

**DOI:** 10.1016/j.vas.2023.100291

**Published:** 2023-03-02

**Authors:** Tatsuro Hifumi, Tetsuya Tanaka, Miho Sato, Kohei Akioka, Chiaki Fujimata, Noriaki Miyoshi

**Affiliations:** a Laboratory of Veterinary Histopathology, Joint Faculty of Veterinary Medicine, Kagoshima University, 1-21-24 Korimoto, Kagoshima 890-0065, Japan; b Laboratory of Infectious Diseases, Joint Faculty of Veterinary Medicine, Kagoshima University, 1-21-24 Korimoto, Kagoshima 890-0065, Japan; c Kumamoto Prefectural Meat Inspection Center, 1314 Sosaki, Shichijo-machi, Kikuchi, Kumamoto 861-1344, Japan

**Keywords:** Alveolar echinococcosis, Horse, Recombinase polymerase amplification, HE, hematoxylin and eosin, Nad5, mitochondrial NADH dehydrogenase subunit 5, PAS, periodic acid-Schiff, PCR, polymerase chain reaction, RPA, recombinase polymerase amplification, LAMP, loop-mediated isothermal amplification

## Abstract

Alveolar echinococcosis in slaughtered horses remains a public health issue. This study aimed to develop a Recombinase polymerase amplification (RPA) assay targeting the mitochondrial NADH dehydrogenase subunit 5 (*Nad5*) gene of *Echinococcus multilocularis* for the rapid detection of equine alveolar echinococcosis. Thirty-six hepatic solid nodules obtained from each horse (*n* = 36) were evaluated based on histopathological examination and *Nad5*-targeted PCR and then submitted to the RPA assay. The results of the developed RPA assay were 94.4% consistent with those of *Nad5* PCR and Cohen's kappa coefficient value was 0.89 statistically, indicating high agreement. In addition, the RPA assay using the plasmid samples was one hundredfold more sensitive than PCR testing. Consequently, these results suggest that the performance of the RPA assay developed in this study is equal to that of conventional PCR testing.

## Highlights

• This is the first study to develop a RPA assay for detecting alveolar echinococcosis in horses.
• The results of the developed LAMP assay on hepatic solid nodules collected from 36 slaughtered horses were 94.4% consistent with those of conventional PCR testing.
• The RPA assay developed in this study is one hundredfold more sensitive than conventional PCR testing.
• The developed RPA assay has a potential alternative to conventional PCR testing to diagnose alveolar echinococcosis in horses.

## Graphical abstract

**Figure:**
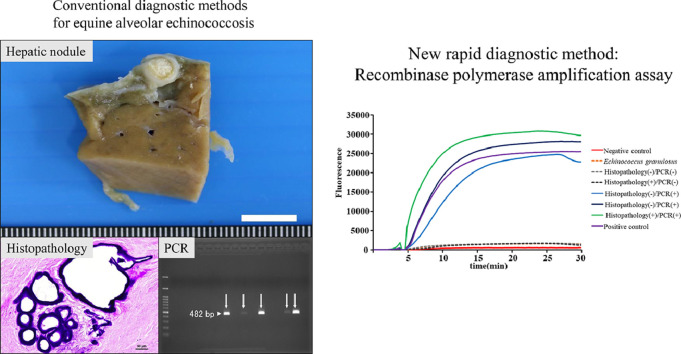

## 1 Introduction

Alveolar echinococcosis caused by larval *Echinococcus multilocularis* infection is a zoonosis of public health importance (Deplazes and Eckert, 2001). *Echinococcus multilocularis* is widely distributed in the Northern Hemisphere, including extensive endemic areas in North America, Europe, and Asia (Eckert and Deplazes, 2004). *E. multilocularis* maintains its life cycle between wild canids (foxes, coyotes, etc.) and dogs as definitive hosts and small rodents (voles, lemmings, etc.) as intermediate hosts (Deplazes and Eckert, 2001; Eckert and Deplazes, 2004). Like humans and pigs, horses are dead-end intermediate hosts of *E. multilocularis* and become infected through the oral ingestion of eggs from definitive host feces (Goto et al., 2010). Lesions caused by larval *E. multilocularis* infection are commonly found in the liver (Goto et al., 2010). Horses may serve as sentinel animals like pigs because they have greater exposure risk of larval *E. multilocularis* infection through eating pasture contaminated by *E. multilocularis* eggs derived from definitive hosts, and their livers can be examined at the time of slaughter (Goto et al., 2010; Hifumi et al., 2015; Tomczuk et al., 2020). Indeed, horse meat inspections in endemic areas such as Japan and Poland have already revealed larval *E. multilocularis* infections in hepatic nodules of slaughtered horses (Goto et al., 2010; Hifumi et al., 2015, 2021a; Tomczuk et al., 2020). In these previous reports, the definitive diagnosis was made based on the results of a histopathological examination and polymerase chain reaction (PCR) testing (Goto et al., 2010; Hifumi et al., 2015, 2021a; Tomczuk et al., 2020). A previous report about using the commercial western blot assay in horses with alveolar echinococcosis has been published (Ueno et al., 2012). As a rapid genetic diagnosis for equine alveolar echinococcosis, a loop-mediated isothermal amplification (LAMP) assay targeting the mitochondrial cytochrome *b* genes of *E. multilocularis* using hepatic nodules of slaughtered horses was developed (Hifumi et al., 2021b). However, histopathological examination and conventional PCR testing usually require time-consuming and complicated experimental manipulation (Hifumi et al., 2021b). Additionally, LAMP assay requires a complicated primer design because the LAMP assay needs four to six primers (Wong et al., 2018).

Although the use of Recombinase polymerase amplification (RPA) assay for the rapid detection of various pathogens including viruses (Abd El Wahed et al., 2013; Yang et al., 2016), bacteria (Eid and Santiago, 2016; Euler et al., 2012; Saxena et al., 2019), parasites (Crannell et al., 2014; Lei et al., 2020, 2019; Sun et al., 2016; Wu et al., 2017) has been reported, there is no information about the detection for *E. multilocularis* infections in definitive and intermediate hosts, including horses. RPA assay is an isothermal amplification technique, performed at 37–42°C, that can amplify as few as 1–10 DNA target copies from 20 min to 40 min (Lobato and O'Sullivan, 2018). The advantage of the RPA assay is that, as in PCR testing, it can be performed with two primers, and, as in the LAMP assay, it can be done under isothermal conditions (Lobato and O'Sullivan, 2018). The mitochondrial NADH dehydrogenase subunit 5 (*Nad5*) gene encoding a protein with moderate to high nucleotide diversity in *Taenia* species is highly suitable for the development of PCR testing and LAMP assay to detect the *Nad5* gene of *E. multilocularis* (Jia et al., 2010; Ni et al., 2014). Based on these backgrounds, it has potential as a targeting gene for the RPA assay. Therefore, this study aimed to develop a RPA assay targeting the *Nad5* gene of *E. multilocularis* to detect alveolar echinococcosis in slaughtered horses with hepatic nodules.

## 2 Material and methods

### 2.1 Field samples

This study was subjected to 36 horses with hepatic nodules that were brought to a slaughterhouse in Kumamoto Prefecture, Japan, between January 2021 and September 2021. These horses showed no clinical signs before slaughter. One nodule from each horse was collected during meat inspection. The biggest nodule was collected if there were multiple nodules in the horse's liver. The nodule size ranged from 1 mm to 25 mm in diameter (Supplementary Table 1). The origins of slaughtered horses were 26 horses from Japan, 9 horses from Canada, and one horse from France (Supplementary Table 1). After bisecting a nodule, one part was examined histopathologically, and the other part was frozen and stored at −20°C for genetic analysis. Nodules were evaluated based on histopathological examination and *Nad5* PCR results. According to a previous report (Hifumi et al., 2021a), cases having laminated layers histopathologically and showing positive for *Nad5* PCR or cases that did not have laminated layers histopathologically and showed positive for *Nad5* PCR were diagnosed as alveolar echinococcosis.

### 2.2 Histopathological examination

Nodules were fixed in 10 or 20% neutral-buffered formalin, decalcified, and neutralized. Then they were embedded in paraffin, sectioned at 3 µm, and submitted to hematoxylin and eosin (HE) stain and periodic acid-Schiff (PAS) reaction.

### 2.3 DNA extraction

A 25 mg tissue sample was collected from each nodule, and the DNA was extracted using a DNeasy Blood and Tissue Kit (Qiagen, Hilden, Germany) according to the manufacturer's instruction.

### 2.4 Conventional PCR testing

Conventional PCR testing was performed to amplify a 482 bp fragment of the *Nad5* gene of *E. multilocularis*. The forward and reverse primers used in this study were designed by Primer3 (https://bioinfo.ut.ee/primer3/) with reference to known base sequences (GenBank accession no. MH259785). The primers are shown in Table 1. The conditions of conventional PCR testing used in this study were as follows; an initial denaturing at 94 °C for 3 min, followed by 35 cycles at 98 °C for 10 s, 60 °C for 15 s, and 68 °C for 45 s, and a final extension at 68 °C for 5 min. An amplification reaction was performed in a total volume of 12.5 µl, containing 1.0 µl of the DNA template, 6.25 µl of 2 × Gflex PCR buffer (Takara Bio Inc., Shiga, Japan), 0.15 µl of each 10 µM primer, 0.15 µl of Tks Gflex DNA polymerase (1.25 U/µl; Takara Bio Inc.), and 4.8 µl of sterile distilled water. A direct sequencing method using a 3730xl DNA Analyzer (Thermo Fisher Scientific, Waltham, Massachusetts, USA) was performed on the PCR products of positive samples to determine the nucleotide sequences. The obtained sequences were compared to sequences registered in the National Center for Biotechnology Information nucleotide database (https://blast.ncbi.nlm.nih.gov/Blast.cgi).

**Table 1:** Designed PCR primers, RPA primers and fluorescent probe targeting the *Nad5* gene of *E. multilocularis*.

**Method**	**Name**	**Sequence (5′→3′)**	**Amplicon Size (bp)**
PCR	PCR-F	ACTGGGTTGTTATTGGTTGGT	482
	PCR-R	AACTAACTGGAGTAGGGGCC	
RPA	RPA-F	TGATTATGTGACGTTTGGTGTGGTAGTTATGC	166
	RPA-R	ATCACCAGTACACACCAAAATACCCATAACAC	
	RPA-P	TATGCTTTTGATATGTTTTTTTTATGTTTA(FAM-dt)T(THF)C (BHQ-dt)ATACTAGACATTATT-SpacerC3	

### 2.5 TA cloning

The PCR-amplified product of the *Nad5* gene obtained from the protoscolex of *E. multilocularis* isolated from Hokkaido was cloned into the T-Vector pMD20 (Takara Bio Inc.) using a Mighty Mix DNA Ligation Kit (Takara Bio Inc.) and transformed into *Escherichia coli* DH5α competent cells. After a properly transformed colony was selected, it was incubated overnight with a Luria-Bertani medium including 50 µg/ml ampicillin. According to the manufacturer's instruction, plasmid DNA was purified from the colony using a QIAGEN Plasmid Mini Kit (Qiagen, Hilden, Germany).

### 2.6 RPA assay

The forward and reverse primers used in the RPA assay were designed by Primer3 (https://bioinfo.ut.ee/primer3/) with reference to known base sequences (GenBank accession no. MH259785). The fluorescent probe was designed according to the manufacturer's instructions for the TwistAmp™ exo kit (TwistDX Inc., Cambridge, UK). Table 1 shows the sequences of the designed RPA primers and the fluorescent probe.

The reactions per four samples using the TwistAmp™ exo kit included 29.5 μl of  rehydration buffer, 12.2 μl of sterile distilled water, 2.1 μl of each 10 μM primer, 0.6 μl of 10 μM fluorescent probe, 2.5 μl of 280 mM magnesium acetate. The DNA template was 0.5 μl per each sample. Amplification was performed using Genelyzer FⅡ (Canon Medical Systems Corporation, Tokyo, Japan) at 39 °C for 30 min. Incubation of samples was performed at the time of 4 min after initiation according to the manufacturer's instructions. Sterile distilled water was used as a negative control.

### 2.7 Assessment of RPA assay compared to conventional diagnostic method from the aspect of analytical sensitivity and statistical analysis

Under the established condition, we performed a RPA assay using DNA obtained from 36 horses with hepatic nodules. Then, we compared the results of the RPA assay with those of histopathological examination and *Nad5* PCR testing to evaluate the diagnostic concordance rate. Cohen's kappa coefficient test was performed using the statistical software EZR ver. 1.61 (https://www.jichi.ac.jp/saitama-sct/SaitamaHP.files/statmedEN.html) to evaluate the degree of agreement statistically (Kanda, 2013). The RPA assay using DNA of *E. granulosus* sensu stricto derived from the liver cyst of cattle was performed to confirm the specificity of the RPA assay developed in this study. The 10-fold serially diluted plasmid samples were subjected to RPA assay to determine the detection limit. The copy number (copies/µl) of the plasmid sample was calculated using the following formula; (Avogadro's number 6.023 × 10^23^/molecular weight of the plasmid sample) × 10^−12^ × concentration of the plasmid sample (pg/µl).

## 3 Results

### 3.1 Summary of the results of histopathology and *Nad5* PCR

In the histopathological examination, 7 of 36 horses (19.4%) had laminated layers in the lesions. In the genetic analysis, 18 of 36 horses (50%), including 6 horses with laminated layers and 12 horses without laminated layers in the lesions, were positive for *Nad5* PCR testing. Therefore, 18 horses were diagnosed with alveolar echinococcosis. A definitive diagnosis could not be made for one horse with laminated layers that were negative for *Nad5* PCR. Seventeen horses lacked laminated layers in the lesions and were negative for *Nad5* PCR. The *Nad5* sequences obtained from all PCR positive samples had 99.58 to 100% identity with those of known *E. multilocularis* (GenBank accession number MH259785). The obtained sequences have already been registered in the database of DNA Data Bank of Japan (https://www.ddbj.nig.ac.jp/index-e.html)(GenBank accession numbers LC756331 and LC756332).

### 3.2 Detection limit of the copy number (copies/µl) using plasmid inserted in the *Nad5* gene

**Fig. 1:**
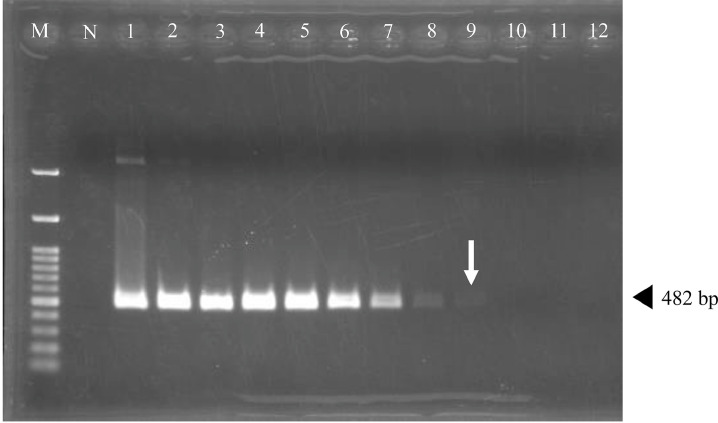
The detection limit of the DNA concentration in the *Nad5* PCR using the 10-fold serially diluted plasmid samples was 3.88 × 10^−5^ pg/µl (sample no.9, white arrow). Lane M: DNA marker 100 bp, lane N: negative control (sterile distilled water), lanes 1–12: 10-fold serial dilutions (1–12: from 3.88 × 10^3^ to 3.88 × 10^−8^ pg/µl).

**Fig. 2:**
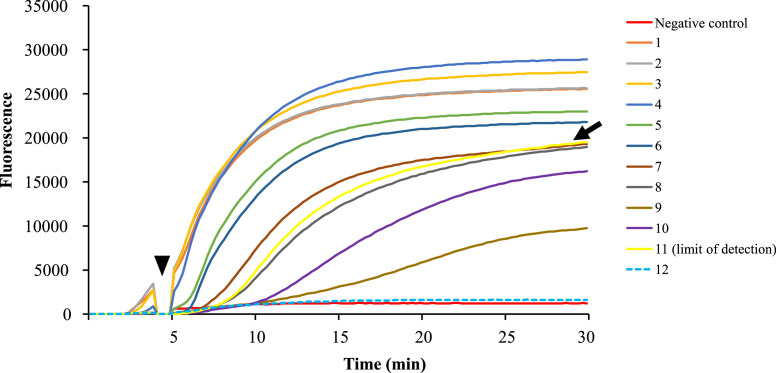
The detection limit of the DNA concentration in the developed RPA assay using DNA derived from 10-fold serially diluted plasmid samples was 3.88 × 10^−7^ pg/µl (sample no.11, black arrow). Samples from Nos.1–12 were 10-fold serial dilutions (1–12: from 3.88 × 10^3^ to 3.88 × 10^−8^ pg/µl). Sterile distilled water was used as a negative control. Incubation of the samples was performed at the time of 4 min after initiation (black arrowhead).

We diluted the plasmid inserted into the *Nad5* gene 10 times serially (from 3.88 × 10^3^ pg/µl to 3.88 × 10^−8^ pg/µl) and performed both PCR testing and RPA assays using these samples. As a result, in PCR testing, the detection limit of the concentration of the plasmid sample was 3.88 × 10^−5^ pg/µl (Fig. 1). Additionally, in the RPA assay, the detection limit of the concentration of the plasmid sample was 3.88 × 10^−7^ pg/µl (Fig. 2). The detection limit of the number of copies was 1 copy/µl. Therefore, the RPA assay developed in this study was one hundredfold more sensitive than conventional PCR testing.

### 3.3 Specificity and sensitivity of the RPA assay on field samples

**Fig. 3:**
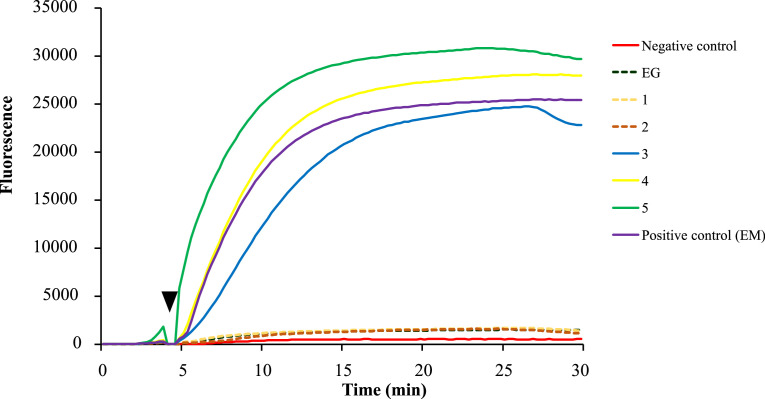
Representative data of the RPA assay using field samples. Sterile distilled water was used as a negative control. DNA derived from the protoscolex of *E. multilocularis* (EM) was used as a positive control. *E. granulosus* sensu stricto DNA derived from the liver cyst of cattle (EG) was used to confirm the specificity of the developed RPA assay. The details of each sample are as follows; 1: Histopathology (−)/PCR (−). Case no. 20, 2: Histopathology (+)/PCR (−). Case no. 19, 3 and 4: Histopathology (−)/PCR (+). Case nos. 7 and 18, respectively, 5: Histopathology (+)/PCR (+). Case no. 2. The abovementioned case numbers correspond with those shown in Supplementary Table 1. Incubation of the samples was performed at the time of 4 minutes after initiation (black arrowhead).

Eighteen horses diagnosed as having alveolar echinococcosis using histopathology and *Nad5* PCR showed positive results in the RPA assay. Additionally, 2 horses that lacked the laminated layers histopathologically and were negative for *Nad5* PCR showed positive results in the RPA assay. Conversely, one horse that had laminated layers histopathologically and was negative for *Nad5* PCR and 15 horses that lacked laminated layers histopathologically and were negative for *Nad5* PCR testing showed negative results in the RPA assay. The sample containing DNA obtained from *E. granulosus* sensu stricto also showed negative for the RPA assay. A representative RPA result for field samples is shown in Fig. 3. Based on the obtained results, the RPA assay has a 94.4% (34/36 horses) concordance rate as compared to conventional PCR results (Table 2). The kappa coefficient value was 0.89 (95%CI: 0.739-1.038), indicating a high degree of statistical agreement. Data including origin, breed, sex, age, nodule size, histopathology, *Nad5* PCR, and RPA results for 36 horses with hepatic nodules are shown in Supplementary Table 1.

**Table 2:** Comparison of the results of RPA assay and histopathology/PCR testing.

	RPA (+)	RPA (−)	Total
Histopathology (+)/PCR (+) (n=6)	6	0	6
Histopathology (−)/PCR (+) (n=12)	12	0	12
Histopathology (+)/PCR (−) (n=1)	0	1	1
Histopathology (−)/PCR (−) (n=17)	2	15	17
Total	20	16	36

## 4 Discussion

The RPA assay showed a 94.4% concordance rate with PCR results. One following possible hypothesis exists for the discrepancy between the results: the RPA assay developed in this study is more sensitive than PCR testing. Comparing the results of PCR testing and the RPA assay using plasmid samples serially diluted 10 times, the RPA assay developed in this study was one hundredfold more sensitive than PCR testing. This result supports the above-mentioned hypothesis. The obtained kappa coefficient value by statistical analysis also demonstrated a high degree of statistical agreement of the results between PCR testing and RPA assay, suggesting the efficacy of the developed RPA assay. In addition, the RPA assay developed in this study did not react to the sample containing *E. granulosus* sensu stricto DNA, which belongs to the same genus *Echinococcus* as *E. multilocularis*. Therefore, the RPA assay designed in this study serves as a more sensitive diagnostic method than conventional PCR testing and can specifically detect equine alveolar echinococcosis in 30 min.

So far, the definitive diagnosis of alveolar echinococcosis in slaughtered horses has been made mainly by conventional PCR testing and histopathological examination (Goto et al., 2010; Hifumi et al., 2015, 2021a; Tomczuk et al., 2020). Compared to PCR testing, the RPA assay has the disadvantage of higher costs for reagents and fluorescent probes. However, in terms of its high sensitivity and easy-to-use operability, the RPA assay for detecting equine alveolar echinococcosis can be a simple and rapid diagnostic method for on-site diagnosis. This is the first report on developing a RPA assay to detect alveolar echinococcosis in horses with hepatic nodules.

## 5 Conclusion

The RPA assay we developed in this study can be used as an alternative to conventional PCR testing for the routine genetic diagnosis of alveolar echinococcosis in horse meat inspection. In addition, as a new rapid genetic diagnostic method, the developed RPA assay can be expected to have applications for epidemiological studies on the definitive hosts of *E. multilocularis* such as wild canids and dogs.

## Ethical statement

The authors declare that an ethical report is not necessary because the present study did not include any experimentation using animals.

## Authors’ contributions

Tatsuro Hifumi and Miho Sato carried out experiments and data analysis. Tatsuro Hifumi drafted the manuscript. Kohei Akioka and Chiaki Fujimata collected the samples. Tetsuya Tanaka and Noriaki Miyoshi reviewed the manuscript. Funding was from Tetsuya Tanaka’s projects. All authors approved the manuscript.

## Funding

This work was supported by the JST Adaptable and Seamless Technology Transfer Program through Target-driven R&D (A-STEP) Grant Number JPMJTM20SV and JSPS KAKENHI Grant Numbers JP20KK0154 and JP22H02522 and HEIWA NAKAJIMA FOUNDATION.

## Supplementary Files

**S. Fig. 1.** Location and sequences of designed PCR primers, RPA primers and fluorescent probe targeting the *Nad5* gene of *E. multilocularis*.

**S. Table 1.** Data for 36 horses with hepatic nodules.

## Declaration of Competing Interest

The authors declare that they have no known competing financial interests or personal relationships that could have appeared to influence the work reported in this paper.

## Appendix Supplementary materials

Image, application 1

Image, application 2
