# Supplementary material for: Rapid detection of alveolar echinococcosis in hepatic nodules of horses by recombinase polymerase amplification assay
Source: Vet Anim Sci. 2023 Mar 2;20:100291. doi: 10.1016/j.vas.2023.100291 (PMC10015249; doi:10.1016/j.vas.2023.100291)
Supplement: Supplementary file 1 [file mmc1.docx]

Supplementary fig 1

>MH259785.1 Echinococcus multilocularis isolate EmXJ NADH dehydrogenase subunit 5 gene, complete cds; mitochondrial

ATGGTTGGTGTTTTTTTTTGTAGTGTAGGTTTGTGTGTGTTGCTATATTGCTTGTTTATTAGTGGTGTTA

GTTATTTGGTTAGGTTTAGGTTTTTATCAATACTGGGTTGTTATTGGTTGGTTAAATTTGATTTTGATTA

TGTGACGTTTGGTGTGGTAGTTATGCTTTTGATATGTTTTTTTTATGTTTATTACTATACTAGACATTAT

TTTGGTGGTTCTTTTTATACTAGGTGTATGTTGTTAAAGTTGGTTGTTTTGTTTGTTAGTGTTATGGGTA

TTTTGGTGTGTACTGGTGATTATTTGTGTACATTGATTTTTTGAGAGTATTTGGGTGTTGTTAGATTTTT

TTTGATTTTGTTTTATGATAGTTTTTTGAGTTTGCGTTCTTCTGTTATTACGTTAGTATCGTCTCGATTT

GGGGATGTGTGCTTGTTTTTATTGATTGGTATAAGTTATTATGTGGATAGTGGGGTATTTCCTTGACTTG

TATGTTTTTTTATGATTGTTTTTTCAAAGAGTGCTGGATTTCCTTTCATTAGTTGATTATTGGAGGCTAT

GCGGGCCCCTACTCCAGTTAGTTCTTTGGTGCATTCTTCAACATTAGTTGCTGCTGGTGTTTGGTTTGTT

ATGCGTTATGATTATTTGTTGCATTTTAGTAGGTCAATAATTATTTTTAGTGTTATGCTTTTGTTGACTG

TGTTTGTAACTGGATTTAGGAGGTTGTTTTTTTATGATTTGAAGAAGATTGTAGCTCTGTCAACATGTAA

TAATGTTTCTTGATGTGTTCTGTACTTGATTTTTGGTGATGTTATGTTGTCGTTGTTTCAATTAATAAGT

CATGGTGTATCTAAGTGTATTTTATTTATGTTAGTTGGTGACGTAATGAGTGGTAGTGGTGGTTCTCAAG

CTAGAAATTGTGTATTTAGATCTCCTTTATATGGATCATGGAATATTTTTGGGTTGTTTGCTGTGGTCCT

TGGTTTAGCTGGAGCTCCATTTATTGGAGTGTTTTTTACTAAGCATTTTTTATTAAGTAGTTTTATTGGT

GTGGTTAGTAATGTTGTGGTTAGTTTGGTTGTTGGAATATGTGTTTTTTTATCGTACTTGTATTCATTTC

GGTTGTGTATTATTTTTTGTACTATAAAGAGTAGATTGTCTTCTGGTGTATTTTTTTATTTTAGTTCAGG

GTTAATGGTGTATTGTTGATTATTTGTTAATTTTTATATATTTTTGCTGTTGAATGAAGTTAATTATTTA

GTTGTGGTTTATAGAGTATTATTAGTTATTTGTCAATTTTTAGCTTTATTATTATCTATTGTATTTTATG

ATAGTTGTGTGTTTAGAATATGAAGTAGTAGGTTATTTGGGTGTGATAATTTAGTTGAATGGTTTTATGA

GCTATTTTATAAGATTTTATTTGTTGTTAATTTTTTTTTTGTTCGGTGGGATTACTTAATGGTTACGTTG

TTTTGTGGTGTTGGTCGTGTTGGTAGAGTAGTTTATGGGTGGATGATGCTTAAAATTTTTTTATTTAGTA

CGTTCGGTTTATTTATATATACATTAGTAGTGTAA

The sequences used for designed primers were colored and the sequences used for the designed fluorescent probe was underlined.

PCR forward primer PCR reverse primer

RPA forward primer RPA reverse primer RPA fluorescent probe
